# Quality of Life After Radical Cystectomy: Meta-analysis of Neobladder and Ileal Conduit Outcomes Across Multiple Assessment Tools

**DOI:** 10.1016/j.euros.2026.03.005

**Published:** 2026-04-16

**Authors:** Ervita Mediana, Agus R.A.H. Hamid, Fakhri Rahman, Chaidir A. Mochtar, Rainy Umbas, Hassan Abol-Enein

**Affiliations:** a Department of Urology, Cipto Mangunkusumo Hospital - Faculty of Medicine, Universitas Indonesia, Jakarta, Indonesia; b Urology and Nephrology Center, Mansoura University, Egypt

**Keywords:** Neobladder, Ileal conduit, Long-term quality of life, Radical cystectomy, Oncology

## Abstract

**Background and objective:**

Radical cystectomy (RC) requires urinary diversion, commonly orthotopic neobladder (ONB) or ileal conduit (IC). While ONB preserves natural voiding, IC is technically simpler. This study aimed to compare long-term (>12 mo) quality of life (QoL) outcomes between ONB and IC to aid preoperative shared decision-making.

**Methods:**

Following PRISMA guidelines, we searched PubMed, Cochrane Library, and Google Scholar up to September 15, 2025. We included studies comparing ONB and IC in adults with follow-up >12 mo. Heterogeneity was explored using meta-regression. The Newcastle-Ottawa Scale assessed bias, and Review Manager v5.4 was used for analysis.

**Key findings and limitations:**

Nineteen studies involving 2379 patients were analyzed. For all assessment tools used (EORTC QLQ-C30, FACT-BL, SF-36, and Bladder Cancer Index [BCI]), higher scores indicate better QoL or function. Pooled analysis showed that ONB was associated with higher global health status (EORTC QLQ-C30: mean difference [MD] = −9.42, *p* = 0.009; negative value indicates higher score in ONB) and functional well-being (FACT-BL −2.60, *p* = 0.010). Conversely, the IC group demonstrated higher scores in urinary outcomes (BCI Urinary: MD = 22.81, *p* = 0.02; positive value indicates higher score in IC). Heterogeneity among studies was moderate to high. Meta-regression indicated geographic location and tumor characteristics influenced heterogeneity. Limitations include observational design and potential selection bias.

**Conclusions and clinical implications:**

ONB reconstruction is associated with higher overall QoL scores, while IC is associated with higher urinary scores. These findings represent clinical trade-offs rather than superiority. Surgical selection should be individualized, balancing patient preference for body image against the risk of functional management challenge.

## Take Home Message

No urinary diversion is universally superior after radical cystectomy; the most appropriate option depends on patient priorities rather than surgical preference alone. The orthotopic neobladder is associated with higher global quality of life and better psychosocial outcomes, largely due to preservation of body image and sexual function. The ileal conduit provides more stable urinary function and simpler daily management, reducing the burden of incontinence and self-care demands. These outcomes represent a clear functional trade-off, not a superiority hierarchy between diversion types. Shared decision-making is essential—selection should incorporate age, comorbidities, lifestyle expectations, and the patient’s willingness and ability to manage long-term self-care requirements.

## 1 Introduction

Radical cystectomy (RC) is a complex surgery that is often chosen for the treatment of high-risk invasive or noninvasive bladder cancer cases [1]. Patients who have undergone RC surgery require urinary tract reconstruction, with neobladder (NB) and ileal conduit (IC) as the main options for urinary diversion [2]. Each method has its benefits and challenges, especially concerning the patient’s long-term quality of life (QoL). The NB method preserves the natural micturition mechanism but is associated with risks of urinary incontinence and metabolic imbalance. Additionally, the IC procedure is a conventional and widely implemented reconstructive option that involves an external urostomy stoma. This adversely affects the patient’s body image and harms the patient’s social functioning [3]. Many studies have compared these two techniques, but the long-term QoL outcomes in patients after RC have not been conclusive. This systematic review and meta-analysis aims to resolve these inconsistencies by focusing specifically on long-term outcomes, defined a priori as a follow-up duration of >12 mo. We hypothesized that while orthotopic NB (ONB) would be associated with more favorable psychological and sexual outcomes potentially linked to body image preservation, IC would be associated with more stable functional metrics in urinary and bowel domains. Unlike previous reviews, this study advances the field by incorporating a meta-regression analysis to explicitly explore study-level sources of heterogeneity, such as geographic region and tumor characteristics. Furthermore, we aim to provide a descriptive synthesis of long-term associations between diversion types and QoL. By rigorously synthesizing evidence from validated instruments, this study seeks to identify patient-centered trade-offs to support preoperative shared decision-making.

## 2 Evidence acquisition

### 2.1 Protocol and registration

This systematic review was conducted in accordance with the PRISMA guidelines (Supplementary Table 1) and adheres to the methodological standards for systematic reviews (MORE) as recommended by the European Urology guidelines. The protocol was prospectively registered in the International Prospective Register of Systematic Reviews (PROSPERO) under the registration number CRD420251116083. Adherence to the methodological standards outlined in the Cochrane Handbook for Systematic Reviews of Interventions was maintained throughout the process.

### 2.2 Search strategy and data sources

A comprehensive and systematic search strategy was devised to identify all relevant literature. We searched three major electronic databases: PubMed/MEDLINE, the Cochrane Library, and Google Scholar. The search window extended from the inception of each database up to September 15, 2025. The search strategy employed a combination of Medical Subject Headings (MeSH) and free-text keywords to maximize sensitivity. Key terms included “Radical Cystectomy,” “Urinary Diversion,” “Orthotopic Neobladder,” “Ileal Conduit,” “Quality of Life,” and validated questionnaire names such as “EORTC QLQ-C30,” “Bladder Cancer Index,” and “FACT-BL.” Boolean operators (AND, OR) were used to refine the search strings (Supplementary Table 2). Additionally, we manually screened the reference lists of included articles and relevant review papers to identify potential studies that might have been missed (“snowballing” technique).

### 2.3 Eligibility criteria

To ensure the validity and comparability of the included data, strict inclusion and exclusion criteria were applied:

1. Population: adult patients (aged >18 yr) diagnosed with bladder cancer who underwent RC.
2. Intervention: construction of an ONB.
3. Comparator: construction of an IC.
4. Outcomes: validated health-related QoL (HRQoL) measures reported as quantitative data (mean and standard deviation [SD]).
5. Follow-up: a minimum follow-up duration of 12 mo was required to exclude acute postoperative recovery issues and focus on long-term survivorship.
6. Study design: randomized controlled trials, prospective cohort studies, retrospective cohort studies, and cross-sectional studies were eligible.
7. Language: only articles published in the English language were included.

Studies without a comparator group, with nonextractable data, or involving nonstandard urinary diversions (eg, continent cutaneous reservoirs) were excluded.

### 2.4 Study selection and quality assessment

Two independent reviewers screened the titles and abstracts of identified records. Potentially relevant full-text articles were retrieved and assessed against the eligibility criteria. Any discrepancies regarding study inclusion were resolved through consensus or consultation with a third senior reviewer.

The methodological quality and risk of bias of the included studies were rigorously assessed using the Newcastle-Ottawa Scale (NOS) for nonrandomized studies. This tool evaluates studies based on three domains:

• Selection: representativeness of the exposed cohort and selection of the nonexposed cohort.
• Comparability: whether the study controls for confounding factors (eg, age, tumor stage).
• Outcome: assessment of outcome independent of the surgeon and adequacy of follow-up. Studies were awarded stars (points), with a maximum score of 9. Studies scoring ≥7 stars were considered high-quality (Supplementary Table 3).

To ensure consistency, data were extracted from the longest follow-up beyond 12 mo reported. In cases where multiple eligible time points were reported (eg, 24, 36, and 60 mo), the longest follow-up data were prioritized to capture sustained survivorship outcomes.

### 2.5 Data extraction and management

Data were extracted using a standardized pilot-tested form. Information collected included: study characteristics (author, year, country, design), patient demographics (sample size, age, gender, body mass index), clinical details (tumor stage, follow-up duration), and outcome data (mean scores and SDs for QoL domains).

A critical methodological decision was made to prioritize postoperative mean scores over pre-post change scores. This approach minimized bias from imputing correlation coefficients for change scores. Where studies reported medians and ranges (or interquartile ranges) instead of means and SDs, we used the robust statistical transformation methods described by Walter and Yao [4] and Wan et al. [5] to estimate the mean and SD (Supplementary Table 4). In instances where a single study reported outcomes using multiple validated instruments (eg, both EORTC QLQ-C30 and Bladder Cancer Index [BCI]) for the same cohort, data were extracted for all relevant domains and analyzed separately within their respective instrument-specific meta-analyses.

### 2.6 Statistical analysis

Quantitative synthesis was performed using Review Manager (RevMan) version 5.4.

• Effect measure: continuous data were analyzed using the mean difference (MD) with 95% confidence intervals (CI).
• Assessment tools orientation: the comparison was structured as IC minus ONB. For all included instruments (EORTC, BCI, FACT-BL, SF-36), higher scores indicate better QoL or function. Consequently, a negative MD indicates higher scores in the ONB group (favoring NB), whereas a positive MD indicates higher scores in the IC group (favoring IC).
• Heterogeneity and model selection: we assessed statistical heterogeneity using the chi-square test and the I^2^ statistic. We anticipated clinical and methodological heterogeneity a priori due to variations in surgical techniques, patient selection, and postoperative protocols across centers. Therefore, a random-effects model (REM) was employed for all primary analyses to provide conservative estimates that account for between-study variance. A fixed-effect model was used only for specific sensitivity analyses where heterogeneity was negligible (I^2^ = 0%) and the underlying assumption of a common effect size was plausible.

### 2.7 Meta-regression analysis

To explore sources of heterogeneity, we conducted random-effects meta-regression analyses. The models examined associations between study-level covariates and the pooled effect sizes (MD). The covariates were defined as follows:

• Age difference: the difference in mean age between the ONB and IC groups (mean age ONB minus mean age IC).
• Sex ratio difference: the difference in the proportion of male participants between groups.
• Geographic region: categorized as European versus non-European centers. These analyses were exploratory and hypothesis-generating, intended to assess ecological associations at the study level rather than causal interactions at the patient level.
• Tumor characteristics: proportion of transitional cell carcinoma (TCC) and pathological stage.

### 2.8 Assessment tools

The QLQ-C30 is one of the most widely used instruments for assessing patient-reported outcomes in oncology [6]. The BCI is a validated bladder cancer–specific tool evaluating urinary, bowel, and sexual function alongside QoL [7]. The SF-36 measures overall well-being across multiple health domains [7], while the FACT-BL specifically assesses QoL in bladder cancer using a 0–4 scale, with higher scores indicating better status [8].

## 3 Evidence synthesis

### 3.1 Search results and study characteristics

**Fig. 1:**
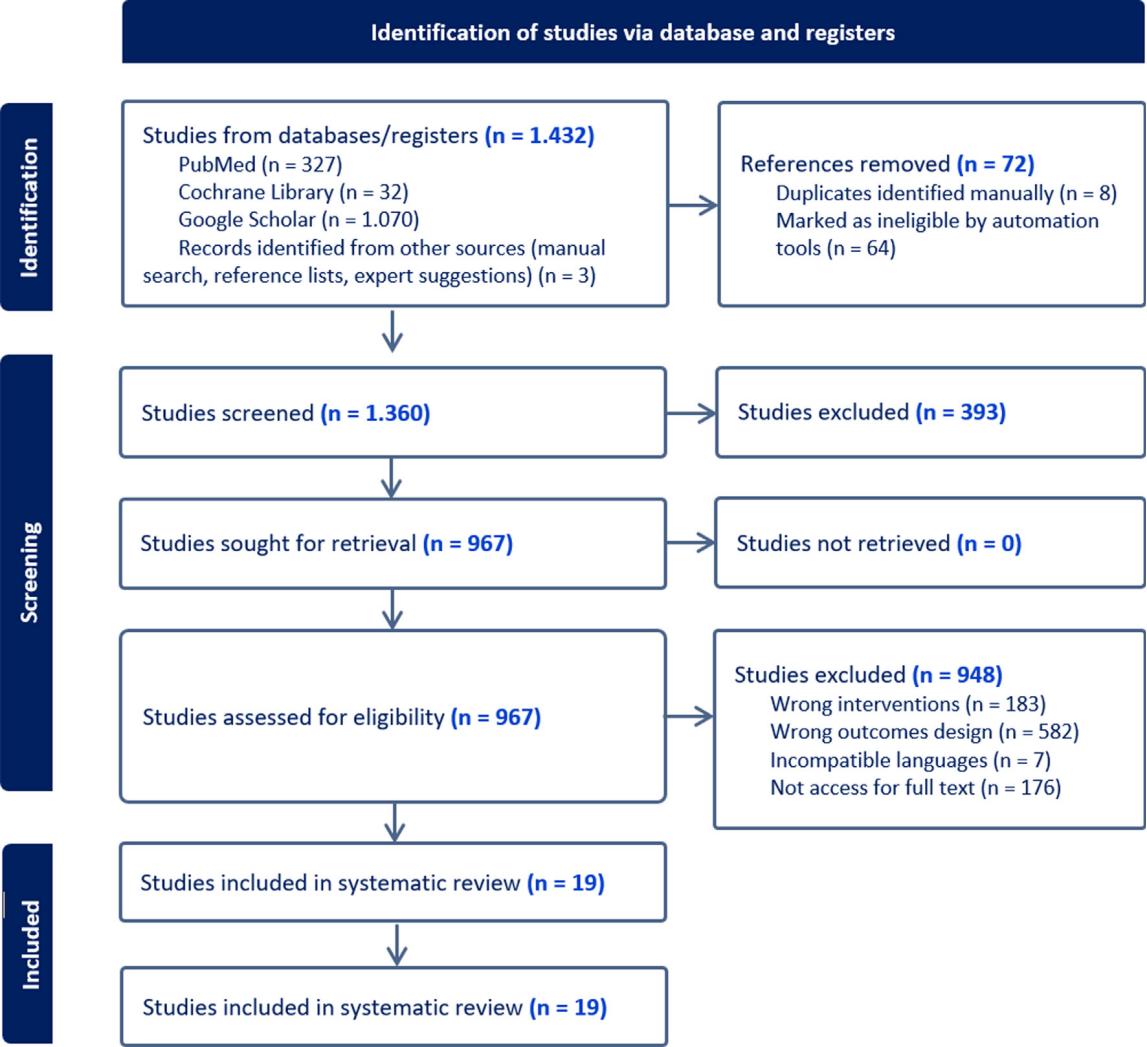
PRISMA flow diagram.

The initial database search yielded 1432 records. After removing duplicates and screening irrelevant titles, 967 full-text articles were assessed. Of these, 19 studies met the strict inclusion criteria and were included in the final analysis (Fig. 1). These 19 studies represent a cumulative sample of 2379 patients, providing a substantial dataset for analysis.

The included studies exhibited a diverse geographic distribution, reflecting global urological practice. We identified cohorts from:

• Europe: Germany (Erber et al. [9], Kretschmer et al. [10]), Italy (Cerruto et al. [3], Siracusano et al. [11], Sogni et al. [12]), France (Biardeau et al. [13]), and the UK (Philip et al. [14]).
• Asia: Japan (Fujisawa et al. [15], Kikuchi et al. [16], Osawa et al. [17]), China (Huang et al. [18]), Thailand (Ditchaiwong et al [19]), and India (Singh et al. [20]).
• Middle East and others: Egypt (Elbadry et al. [8], Mahmoud et al [21], Zahran et al. [22]) and the USA (Gellhaus et al. [2], Reed et al. [23]).

The study designs were mixed, comprising five prospective cohort studies, four retrospective analyses, and 10 cross-sectional surveys. Importantly, most studies reported follow-up durations significantly exceeding the 12-mo minimum threshold, with several studies reporting outcomes at ≥5 yr, offering true long-term insights. A summary data is provided in Table 1.

**Table 1:** Baseline characteristics of included studies

No	Study ID, author, yr (country)	Study design	Sample size, (ONB/IC)	Follow-up duration	Population characteristics			Tumor characteristics			Surgical procedure characteristics
					Sex (male/female) [ONB//IC]	Mean age (SD) [ONB/IC]	Mean BMI [ONB/IC]	TCC [ONB/IC]	Grade pT ≥3 [ONB/IC]	Grade pN ≥1 [ONB/IC]	
EORTC QLQ-C30											
1	Biardeau et al., 2020 (France) [13]	Cross-sectional study	17/23	>24 mo	All female populations	60.7 (7)/65.8 (11.8) a	23.7 (2.6)/21.3 (8.9) a	16/21	5/10	2/5	Not specified
2	Cerruto et al., 2017 (Italy) [3]	Cross-sectional study	171/148	>24 mo	156/15//115/33	64.3 (9.4)/70.8 (8.3)	26.9 (3.2)/25.9 (3.4)	NI	21/21	21/21	Not specified
3	Erber et al., 2012 (Germany) [9]	Retrospective study	115/146	>24 mo	110/5 // 98/48	62 (2.5)/70 (2.8)	26.1 (3.1)/25.9 (4.2)	52/47	17/21	12/10	Not specified
4	Kretschmer et al., 2019 (Germany) [10]	Prospective	67/67	>24 mo	53/14 // 50/17	67.8 (9.9)/68.4 (10.3)	26.6 (4.8)/26.4 (4.5)	67/67	25/27	NI	Hautmann neobladder
5	Singh et al., 2013 (India) [20]	Prospective	80/84	12–24 mo	69/11//74/10	58.7 (8.9)/56.1 (7.3)	24.3 (2.1)/23.8 (2.0)	75/82	5/9	8/6	Not specified
6	Sogni et al., 2008 (Italy) [12]	Retrospective	32/53	>24 mo	NI	78 (1.8)/80.2 (3.3)	NI	NI	NI	NI	Not specified
7	Zahran et al., 2017 (Egypt) [22]	Cross-sectional	84/61	>24 mo	All female populations	NI	NI	NI	NI	NI	Serous-lined extramural tunnel (52), ileal W neobladder/ Hautmann (28), and kock ileal neobladder (3). Surgical without otonom nerve preservation
8	Siracusano et al., 2019 (Italy) [11]	Cross-sectional	58/64	12–24 mo	51/7//45/19	68 (8)/73 (7)	26 (3)/27 (4)	58/64	18/27	11/11	Not specified
FACT-BL											
9	Ditchaiwong et al., 2023 (Thailand) [19]	Cross-sectional	18/60	12–24 mo	18/0//45/15	62.4 (8.2)/67.2 (8.8) a	NI	NI	6/32	2/6	Studer neobladder (ONB) Ileal conduit (IC)
10	Elbadry et al., 2020 (Egypt) [8]	Prospective	49/64	12–24 mo	44/5//53/11	59.3 (8.3)/61.9 (7)	28 (6.5)/27.1 (5.3)	36/58	21/32	NI	Not specified
11	Kikuchi et al., 2006 (Japan) [16]	Cross-sectional	15/20	>24 mo	15/0//13/7	63.6 (5.5)/66.8 (10.3)	NI	NI	3/5	NI	Not specified
12	Mahmoud et al., 2019 (Egypt) [21]	Retrospective	36/39	12–24 mo	36/4//39/7	60.7 (6.8)/62.4 (5.8)	25.6 (2.2)/IC 25.3 (3)	NI	15/20	6/6	Not specified
12	Zahran et al., 2017 (Egypt) [22] 2017 (Same as study number 7)	Cross-sectional study	84/61	>24 mo	All female populations	52.5 (2.8)/51.5 (2.8)	NI	NI	NI	NI	Serous-lined extramural tunnel (52), ileal W neobladder/ Hautmann (28), and kock ileal neobladder (3). Surgical without otonom nerve preservation
SF-36											
13	Fujisawa et al., 2000 (Japan) [15]	Retrospective	36/20	>24 mo	26/10//12/8	63.5 (7.9)/73.5 (8.9)	NI	NI	NI	NI	Not specified
14	Philip et al., 2009 (UK) [14]	Cross-sectional	28/24	12–24 mo	25/3//15/9	NI	NI	NI	NI	NI	Not specified
BCI											
15	Gellhaus et al., 2016 (US) [2]	Cross-sectional	48/44	>24 mo	47/1//35/9	58.4 (9.1)/67.2 (9.4)	NI	NI	9/13	7/6	Not specified
16	Goldberg et al., 2015 (Israel) [24]	Cross-sectional	46/49	>24 mo	44/2//44/5	61 (7.8)/57.2 (9.8) a	NI	NI	11/11	8/3	Not specified
17	Huang et al., 2015 (China) [18]	Retrospective	39/78	>24 mo	34/5//68/10	63.6 (6.1)/64 (5.7)	NI	NI	6/14	0/2	Hautmann technique
18	Osawa et al., 2021 (Japan) [17]	Cross-sectional	49/101	>24 mo	NI	NI	NI	NI	NI	NI	Not specified
19	Reed et al., 2019 (US) [23]	Cross-sectional	54/66	>24 mo	48/6//46/20	59.8 (9.6)/69.3 (9.1)	NI	NI	NI	NI	Not specified

NI = no information.

a Requires data conversion and estimation to mean (SD).

### 3.2 Global health status (EORTC QLQ-C30)

The EORTC QLQ-C30 is the most widely used generic cancer instrument. Eight studies contributed data to the global health status (GHS) domain. The pooled random-effects analysis demonstrated a significant benefit in favor of the ONB (a summary of all pooled estimates is provided in Supplementary Table 1), with a MD of −9.42 (95% CI: −16.46 to −2.37; *p* = 0.009); as the comparison was conducted between IC and ONB, this negative value confirms that patients undergoing ONB reported significantly higher global health and overall QoL scores than those receiving IC. However, this finding must be interpreted in the context of high heterogeneity (I^2^ = 86%). To dissect this, we performed subgroup analysis by study design:

• Prospective studies: consistently favored the ONB (MD = −17.89, 95% CI: −22.41 to −13.38), reinforcing the finding that ONB is associated with better global health scores even in prospectively followed cohorts.
• Retrospective/cross-sectional studies: Tended to favor ONB, which may reflect a “survivorship bias” where patients with successful NBs are more likely to participate in surveys years later.

To strictly evaluate the robustness of these findings against methodological flaws, a dual-criteria sensitivity analysis was performed. We excluded studies that used data imputation (converting medians/ranges to means) and studies with a high risk of bias (NOS score <5). This stringent exclusion removed Zahran et al, Biardeau et al, and Kretschmer et al. The resulting pooled estimate from the remaining high-quality studies (*n* = 5) showed a MD of −8.14 (95% CI: −19.05 to 2.77; *p* = 0.14). While statistical significance was impacted by the reduced sample size, the direction and magnitude of the association remained remarkably consistent with the primary analysis (MD = −9.42), suggesting that the trend favoring ONB is not an artifact of low study quality or data imputation (see Table 2).

**Table 2:** Summary of reported quality of life outcomes (mean ± SD)

EORTC QLQ-C30							
No	Study ID	Global health status [ONB/IC]	Physical functioning [ONB/IC]	Role functioning [ONB/IC]	Emotional functioning [ONB/IC]	Cognitive functioning [ONB/IC]	Social functioning [ONB/IC]
1	Biardeau et al., 2020 [13]	70.8 (20.8)/66.7 (16.7)^a^	81.6 (15)/70 (13)^a^	75 (25)/79.2 (20.8)^a^	72.9 (22.9)/70.8 (20.8)^a^	76.7 (23.3)/68.7 (22.9) a	75 (25)/25 (25) a
2	Cerruto et al., 2017 [3]	64.9 (25.2)/62.3 (25.1)	80.8 (22)/74.7 (24.8)	72.1 (32.7)/76.9 (29)	84.9 (20.9)/78.7 (24.7)	93.1 (12.6)/85.4 (21.2)	80.8 (24.6)/83.6 (23)
3	Erber et al., 2012 [9]	72.3 (19.5)/58.3 (25.3)	82.6 (19.9)/65.8 (29.4)	76 (27.9)/63.8 (13.1)	81.1 (22.3)/72.2 (22.3),	83.3 (20.5) /77.8 (22.9)	70.1 (33)/65.3 (32.2)
4	Kretschmer et al., 2019 [10]	73.6 (19.3)/60.5 (24.6)	83.5 (19.8)/64.5 (29.3)	76.4 (26.2)/60.3 (34.8)	79.9 (21.9)/76.2 (21)	86.6 (23.5)/85.8 (19.3)	79.6 (25.6)/65.7 (32.8)
5	Singh et al., 2013 [20]	88 (6.6)/70 (10.5)	75.3 (12)/76 (8.6)	95.2 (8.1)/76.6 (8.6)	77.3 (6.3)/76.6 (16.1)	78.5 (23)/76.6 (16.1)	92.8 (8.9)/76.6 (8.6)
6	Sogni et al., 2008 [12]	77 (26.9)/65 (20.9)	78 (27.1)/78 (21.7)	83 (27.8)/83 (24.9)	89 (17.9)/87 (14.1)	89 (21.7)/90 (13)	90 (27.1)/83 (22.9)
7	Zahran et al., 2017 [22]	67.7 (18.9)/52.1 (22.9)	57.71 (23.6)/60 (20)^a^	61 (22.1)/66 (25)^a^	57.8 (29.1)/75 (25)^a^	63.1 (30.4)/74 (19.5) a	58.6 (29.3)/66 (25) a
8	Siracusano et al., 2019 [11]	72 (20)/61 (22)	88 (15)/76 (20)	85 (23)/67 (32)	84 (19)/74 (25)	88 (16)/80 (23)	85 (23)/69 (30)
FACT-BL							
No	Study ID	Total FACT-BL [ONB/IC]	Physical well-being [ONB/IC]	Social/family well-being [ONB/IC]	Emotional well-being [ONB/IC]	Functional well-being [ONB/IC]	
9	Ditchaiwong et al., 2023 [19]	109.8 (18.1)/102.5 (17.6)^a^	25.2 (2.6)/23.4 (4.3)^a^	23.2 (2.9)/23.3 (4.2)^a^	21.1 (3.4)/19.3 (3.7)^a^	19.8 (5.2)/17.2 (5.4) a	
10	Elbadry et al., 2020 [8]	82.4 (7.4)/88.9 (7.7)	13.7 (1.4)/15 (1.6)	21.8 (1.8)/21.7 (1.8)	15.3 (3.9)/14.5 (4)	16.2 (3.3)/14.4 (3.2)	
11	Kikuchi et al., 2006 [16]	110.9 (18)/106.3 (16.4)	26.6 (3.4)/24.5 (3.5)	17.2 (8.4)/17.9 (5.6)	21.1 (2.8)/18.5 (4.9)	20.5 (9.3)/20.2 (6.3)	
12	Mahmoud et al., 2019 [21]	134.4 (6.3)/105 (10.2)	25.5 (2)/17.6 (3.2)	23.7 (1.3)/19.2 (1.9)	22.1 (1.2)/16.9 (2.4)	23.9 (1.5)/18.9 (1.7)	
12	Zahran et al., 2017 [22](Same as study number 7)	95.5 (18.2)/89.9 (22.6)^a^	16.6 (5.4)/14 (6.8)^a^	17.8 (6.01)/18 (6.5)^a^	16.3 (5.1)/13 (6)^a^	16.8 (6.6)/15 (5)^a^	
SF-36							
No	Study ID	Global health [ONB/IC]					
13	Fujisawa et al., 2000 [15]	56.9 (18.5)/64.9 (18.9)					
14	Philip et al., 2009 [14]	73.8 (20)/68.2 (22)					
BCI							
No	Study ID	Urinary functioning [ONB/IC]	Bowel functioning [ONB/IC]	Sexual functioning [ONB/IC]			
15	Gellhaus et al., 2016 [2]	30.6 (79.5)/79.5 (26.8)	82.6 (15.3)/79.9 (14.7)	30.7 (25.8)/18 (21.7)			
16	Goldberg et al., 2015 [24]	64.7 (14.4)/83.3 (12)^a^	53 (11)/55.5 (9)^a^	38.8 (21.6)/26.1 (20.4)^a^			
17	Huang et al., 2015 [18]	75 (4)/82 (5)	NI	NI			
18	Osawa et al., 2021 [17]	52.5 (2.8)/89.2 (2.1)^a^	76.6 (2.7)/85.6 (1.9)^a^	6.9 (2.8)/5.1 (2)^a^			
19	Reed et al., 2019 [25]	NI	55.4 (12.4)/56.4 (13.7)	40.8 (28.7)/25.1 (24.4)			

NI = no information.

a Requires data conversion and estimation to mean (SD).

### 3.3 Condition-specific outcomes: The BCI

The BCI is highly sensitive to the functional nuances of urinary diversion. Our analysis of this instrument revealed the most distinct “trade-offs” between the two techniques (a summary of all pooled estimates is provided in Supplementary Table 1).

• Urinary function: the pooled analysis strongly favored the IC (MD = 22.81, 95% CI: 2.99 to 42.64, *p <* 0.00001). This substantial difference highlights the functional stability of the IC. IC patients typically report “normal” function because the stoma bags work passively. In contrast, ONB patients scored lower due to issues such as daytime incontinence (requiring pads), nocturnal enuresis, and the need for timed voiding.
• Bowel function: regarding bowel function, the analysis did not observe a statistically significant difference between groups (MD = 2.86, 95% CI: −2.94 to 8.66, *p* = 0.33). Although the IC group had numerically higher scores, the outcomes were statistically comparable to the NB group.
• Sexual function: in contrast, the meta-analysis assessing sexual function demonstrated no statistically significant difference between groups (MD = −2.23, 95% CI: −15.04 to 10.59; *p* = 0.73). This analysis was accompanied by substantial heterogeneity. Interpretation of these findings is further limited by the small number of studies evaluating sexual function, which reduces the robustness of the pooled estimate. This finding was consistent across studies (Gellhaus et al. [2], Goldberg et al. [24], Osawa et al. [17]**)**.

For the BCI domain, a sensitivity analysis based on risk of bias (NOS score) was not feasible as it would have resulted in insufficient studies for pooling (k *<* 2). Therefore, the sensitivity analysis was restricted to the exclusion of studies using data imputation (Osawa et al and Goldberg et al) revealed notable shifts in the BCI domains. For urinary function, the exclusion of these studies resulted in a loss of statistical significance (MD = 17.42, 95% CI: −4.48 to 39.31; *p* = 0.12), although the direction of effect still favored the IC. Remarkably, for sexual function, the sensitivity analysis revealed a statistically significant advantage for the NB (MD = −14.21, 95% CI: −21.06 to −7.36; *p <* 0.0001) with heterogeneity dropping to 0%. This suggests that while the overall pooled analysis was diluted by heterogeneous imputed data, the subset of studies reporting direct MDs/SDs indicates a strong potential benefit for ONB in sexual outcomes.

### 3.4 Other instruments (FACT-BL and SF-36)

Analysis of the FACT-BL questionnaire echoed the findings of the EORTC QLQ-C30. The total score significantly favored ONB, particularly in the “Functional Well-Being” (FWB) subdomain. Interestingly, the SF-36 “General Health” domain did not show a statistically significant difference between groups (MD = 1.50, 95% CI: −11.81 to 14.82, *p* = 0.83), suggesting that from a broad, noncancer-specific perspective, overall physical health is comparable regardless of the diversion type [25].

A sensitivity analysis was conducted by excluding studies that used data transformation (Zahran et al. and Ditchaiwong et al.). Notably, the remaining studies included in this sensitivity analysis all demonstrated low risk of bias (NOS score >5). Consequently, the resulting estimates reflect a synthesis of high-quality primary data. The analysis showed consistent effect sizes, particularly in the FWB domain, where the MD remained stable at −2.87 (95% CI: −5.71 to −0.03; *p* = 0.05), compared to −2.60 in the primary analysis. This suggests that the favorable association with ONB in FWB is consistent across high-quality data and is not an artifact of data imputation.

### 3.5 Meta-regression analysis

Our meta-regression analysis provided insights into the heterogeneity observed (Table 3).

• Geographic influence: we observed that EORTC QLQ-C30 outcomes varied by geographic region at the study level (*p* = 0.0064). Studies conducted in Europe reported GHS scores approximately 9 points lower than studies from non-European regions (Asia/United States).
• Tumor factors: the proportion of TCC pathology was also associated with the degree of between-study heterogeneity (*p* = 0.0087).
• Demographics: we did not find evidence that the ONB–IC differences varied by study-level age or gender composition.

**Table 3:** Summary of meta regression

Moderator	β (Estimate)	SE	z	*p* value	95% CI (lower)	95% CI (upper)	k (studi)	R^2^ (%)	Brief interpretation
EORTC QLQ-C30									
Male proportion (centered)	0.42	0.63	0.66	0.51	–0.82	1.66	7	0%	We did not observe a statistically significant association between the proportion of male patients and the effect size
Age (mean)	−0.06	0.04	−1.66	0.10	−0.13	0.01	7	29.04%	Older age showed a trend toward a smaller effect, but this association was not statistically significant (*p* = 0.097)
BMI (mean)	3.29	0.20	−0.51	0.61	−0.49	0.29	6	0%	BMI was not significantly associated with effect size variation in this model.
Difference in TCC proportion	−4.10	14.65	−0.28	0.78	−32.82	24.63	5	0%	No significant association was found between TCC proportion differences and QoL outcomes.
Different in pT stage proportion	-5.80	24.62	−0.24	0.81	-54.06	42.46	6	0%	No significant effect of differences in pT proportion on the outcome.
Different in pN stage proportion	7.58	6.71	1.13	0.26	-5.57	20.73	5	7.18	Differences in pN stage proportion were not significantly associated with the outcome.
Proportion of TCC (ONB group)	−171.8	65.45	−2.63	0.01	−300.12	−43.56	5	100	A higher proportion of TCC in the ONB group was associated with lower GHS scores.
Proportion of TCC (IC group)	134.74	50.57	2.66	0.01	35.63	233.85	5	100	A higher proportion of IC in the ONB group was associated with lower GHS scores.
Geographic Region (Europe vs. Non-Europe)	−9.05	3.32	−2.73	0.01	−15.57	−2.54	8	72.87	Geographic region was a significant moderator; European studies were associated with lower GHS scores compared to non-European studies.
FACT-BL									
Male proportion (centered)	−3.21	16.58	−0.19	0.85	−35.70	29.29	5	0	We did not observe a statistically significant association between the proportion of male patients and the effect size.
Age (mean)	1.34	1.78	0.75	0.45	−2.14	4.82	4	23.31	Age difference between the ONB and IC groups was not significantly associated with effect size variation in this model.
BCI									
Male proportion (centered)	2.64	1.66	1.59	0.11	−0.62	5.90	3	100	We did not observe a statistically significant association between the proportion of male patients and the effect size.
Age (mean)	−0.04	0.03	−1.41	0.16	−0.08	0.01	3	100	Age difference between the ONB and IC groups was not significantly associated with effect size variation in this model.

TCC = transitional cell carcinoma; pT = pathological tumor stage; pN = pathological nodal stage.

## 4 Discussion

### 4.1 Interpreting the “trade-off” hypothesis

The findings of this meta-analysis provide strong empirical support for the “trade-off” hypothesis in urinary diversion. There is no single “winner”; rather, each diversion type excels in different domains. Regarding sexual function, our pooled analysis did not demonstrate a statistically significant advantage for ONB, contradicting the assumption that body image preservation automatically translates to better sexual outcomes. While some individual studies favor ONB, the high heterogeneity likely reflects variations in nerve-sparing techniques, patient age, and baseline function across cohorts, preventing a definitive conclusion of superiority [25].

Conversely, the IC excels in functional reliability. The lower urinary function scores in ONB patients reflect the burden of “NB management.” Unlike the passive drainage of an IC, an ONB requires active patient participation timed voiding every 3–4 h, pelvic floor muscle training, and vigilance against mucus obstruction [24]. Furthermore, nocturnal enuresis remains a persistent issue for many ONB patients, often requiring them to wake up multiple times at night or wear protective pads. For patients who prioritize uninterrupted sleep and simplicity of care, the IC may represent a more favorable option associated with greater functional stability [26].

### 4.2 Impact of selection bias and confounding by indication

A major challenge in interpreting observational data is confounding by indication. In real-world clinical practice, the allocation of urinary diversion is never random. Patients selected for ONB are systematically “better” candidates; they are younger, have fewer comorbidities (lower ASA scores), better renal function, and higher cognitive motivation compared to those receiving an IC. Our review of baseline characteristics confirms this, with ONB cohorts consistently being younger than their IC counterparts (eg, in Cerruto et al, age was 64 vs 71 yr).

The higher GHS observed in the ONB group likely reflects both diversion-related factors and more favorable baseline patient characteristics. Despite use of random-effects models, residual confounding remains, and the QoL benefits of ONB may not be generalizable. Clinicians should thus recognize that older or frailer patients undergoing ONB may not experience the same QoL benefits as the younger, healthier cohorts [3].

Beyond statistical significance, it is essential to interpret these findings in terms of clinical relevance. The pooled MD of −9.42 points in the GHS domain approaches the upper limit of the established minimal clinically important difference for the EORTC QLQ-C30, which is commonly defined as a change of 5–10 points [27]

This indicates that the statistical advantage of ONB reflects a clinically meaningful improvement in daily QoL rather than a trivial numerical difference driven solely by large sample sizes.

### 4.3 Regional variations and cultural context

The finding from our meta-regression that European studies report lower QoL scores is a novel and important contribution. This regional discrepancy may stem from several factors. First, the observed regional disparities may reflect cultural differences in how health and illness are expressed, or variations in patient behaviors. Second, differences in health care delivery such as the intensity of postoperative rehabilitation or the availability of stoma nurses may vary by region. This underscores the need for culturally sensitive counseling; data derived from a United States or Asian cohort may not be perfectly applicable to a European patient, and vice versa [10].

### 4.4 Surgical era effects and technical evolution

The studies included in this review span a period of significant surgical evolution, from the early 2000s to 2025. This introduces an “era effect.” Early studies (eg, Fujisawa et al. [15]) reflect the outcomes of open RC with older NB configurations. More recent studies (eg, Kretschmer et al [10]; Osawa et al. [17]) capture the era of Robotic-Assisted Radical Cystectomy (RARC) and Enhanced Recovery After Surgery protocols. RARC may reduce morbidity in ONB construction, contributing to faster recovery and improved early QoL. Advances in nerve-sparing techniques may further enhance sexual function outcomes of ONB. Future studies should stratify results by surgical approach (open vs robotic).

### 4.5 Clinical implications for shared decision-making

The data derived from this study can contribute to the preoperative counseling process. The “ideal” urinary diversion does not exist; the “right” diversion is the one that aligns with the patient’s specific values and lifestyle.

• Patient profile A (ONB candidate): a younger, socially active patient who prioritizes body image, wishes to conceal their surgery. This patient must be willing to accept the “job” of managing an NB (timed voiding, potential leakage) to gain these psychological benefits.
• Patient profile B (IC candidate): an older patient, or one with limited manual dexterity or cognitive reserve, who prioritizes sleep quality, simplicity of care, and avoidance of catheterization risk. For this patient, the IC is not a “suboptimal” choice but a valuable option associated with greater functional stability.

Clinicians should use the BCI domain scores presented here to set realistic expectations: telling ONB candidates that while their *sexual function* may be better preserved, their *urinary control* will statistically be inferior to an IC, at least in terms of pad usage and nighttime dryness.

### 4.6 Limitations

Despite rigorous methodology, this study has several limitations. First, the predominance of cross-sectional designs introduces survivorship bias; patients with failed NBs (converted to conduits) are often excluded from long-term surveys, potentially artificially inflating ONB scores. Second, the pooled estimates exhibited high heterogeneity (I^2^ > 80%), which reflects variability in unmeasured confounding factors such as surgeon volume, surgical techniques, and institutional rehabilitation protocols across centers. Third, the inclusion of Google Scholar as a data source, without strictly predefined screening thresholds for grey literature, may have introduced a potential selection bias. Finally, defining “long-term” as >12 mo aggregates heterogeneous follow-up durations, potentially obscuring functional changes over long-term survivorship.

## 5 Conclusions

This systematic review and meta-analysis suggests distinct QoL trade-offs between urinary diversion types. ONB reconstruction is associated with higher scores in GHS, whereas IC diversion is associated with more favorable scores in urinary domains. However, given the substantial heterogeneity and inherent selection bias in the included observational studies, these findings do not establish the definitive superiority of one technique. Instead, clinical practice should prioritize shared decision-making, weighing the patient’s preference for body image preservation against the need for functional stability.

  ***Authors contributions***: Ervita Mediana had full access to all the data in the study and takes responsibility for the integrity of the data and the accuracy of the data analysis.

  *Study concept and design:* Mediana.

*Acquisition of data:* Mediana, Taher.

*Analysis and interpretation of data:* Mediana, Hamid, Taher.

*Drafting of the manuscript:* Mediana, Taher.

*Critical revision of the manuscript for important intellectual content:* Hamid, Mochtar, Umbas, Enein.

*Statistical analysis:* Mediana, Hamid, Taher.

*Obtaining funding:* None.

*Administrative, technical, or material support:* None.

*Supervision:* Hamid, Mochtar, Umbas, Enein.

  ***Financial disclosures:*** Ervita Mediana certifies that all conflicts of interest, including specific financial interests and relationships and affiliations relevant to the subject matter or materials discussed in the manuscript (eg, employment/affiliation, grants or funding, consultancies, honoraria, stock ownership or options, expert testimony, royalties, or patents filed, received, or pending), are the following: None.

  ***Funding/Support and role of the sponsor****:* None.

  ***Acknowledgments:*** The authors thank Pedro Markus Sanggara Purba for the support in data analysis.
