# Supplementary material for: Quality of Life After Radical Cystectomy: Meta-analysis of Neobladder and Ileal Conduit Outcomes Across Multiple Assessment Tools
Source: Eur Urol Open Sci. 2026 Apr 16;87:115–24. doi: 10.1016/j.euros.2026.03.005 (PMC13101609; doi:10.1016/j.euros.2026.03.005)
Supplement: Supplementary Data 1 [file mmc1.docx]

Supplementary Material

1. **Supplementary Figure 1a:** Forest Plot for EORTC QLQ-C30 Global Health Status.


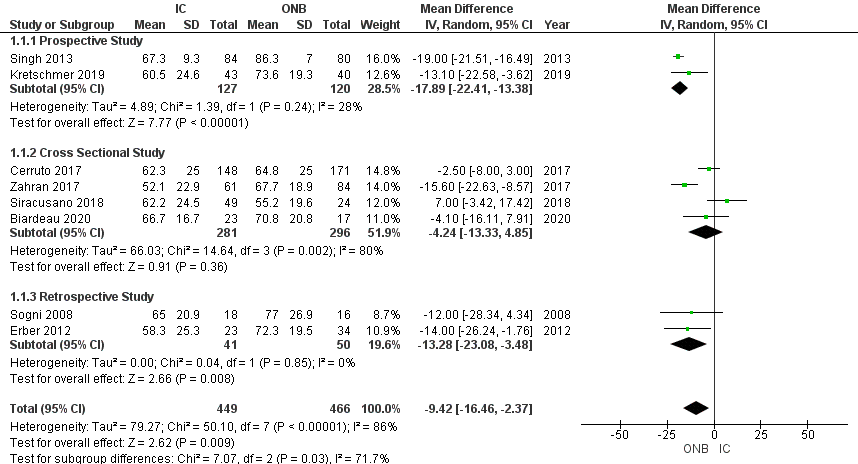


Note: The analysis compares Ileal Conduit (IC) versus Orthotopic Neobladder (ONB). Results are presented as Mean Differences (MD). Since higher scores indicate better quality of life, a negative MD value (< 0) favors the Neobladder group, while a positive MD value (> 0) favors the Ileal Conduit group. Horizontal bars denote 95% Confidence Intervals (CIs).

**2.**  **Supplementary Figure 1b:** Forest Plot for EORTC QLQ-C30 Global Health Status after Sensitivity Analysis


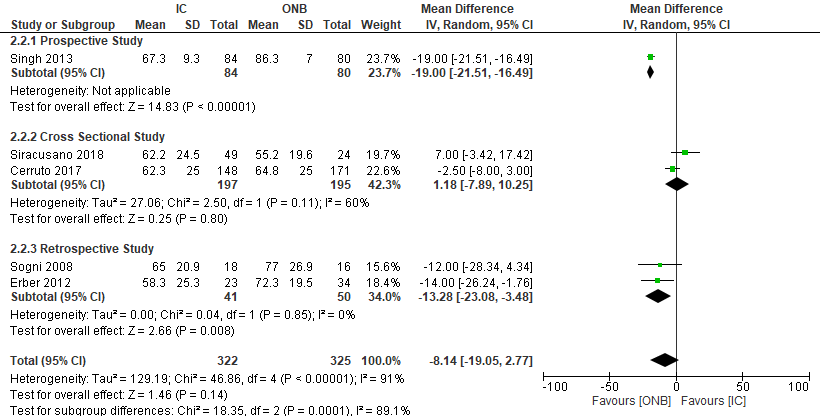


Note: The analysis compares Ileal Conduit (IC) versus Orthotopic Neobladder (ONB). Results are presented as Mean Differences (MD). Since higher scores indicate better quality of life, a negative MD value (< 0) favors the Neobladder group, while a positive MD value (> 0) favors the Ileal Conduit group. Horizontal bars denote 95% Confidence Intervals (CIs).

**3. Supplementary Figure 2:** Forest Plot for EORTC QLQ-C30 Physical Functioning.

**
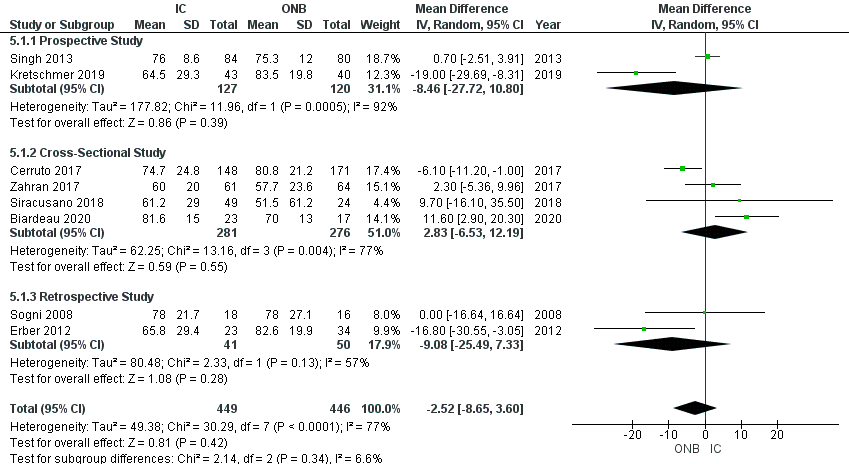
**

Note: The analysis compares Ileal Conduit (IC) versus Orthotopic Neobladder (ONB). Results are presented as Mean Differences (MD). Since higher scores indicate better quality of life, a negative MD value (< 0) favors the Neobladder group, while a positive MD value (> 0) favors the Ileal Conduit group. Horizontal bars denote 95% Confidence Intervals (CIs).

**4. Supplementary Figure 3a:** Forest Plot for BCI Functional Outcomes (Urinary, Bowel, Sexual).


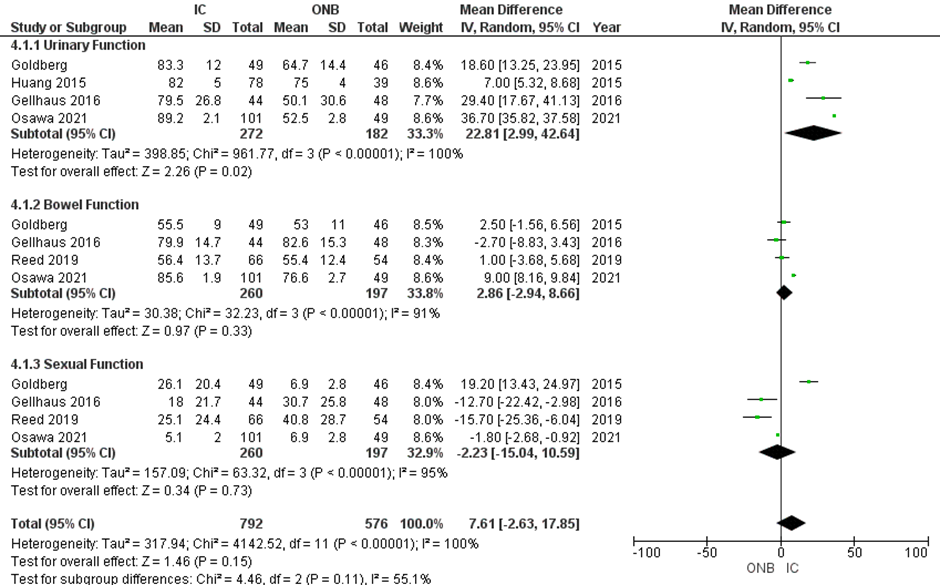


Note: The analysis compares Ileal Conduit (IC) versus Orthotopic Neobladder (ONB). Results are presented as Mean Differences (MD). Since higher scores indicate better quality of life, a negative MD value (< 0) favors the Neobladder group, while a positive MD value (> 0) favors the Ileal Conduit group. Horizontal bars denote 95% Confidence Intervals (CIs).

**5. Supplementary Figure 3b:** Forest Plot for BCI Functional Outcomes After Sensitivity Analysis


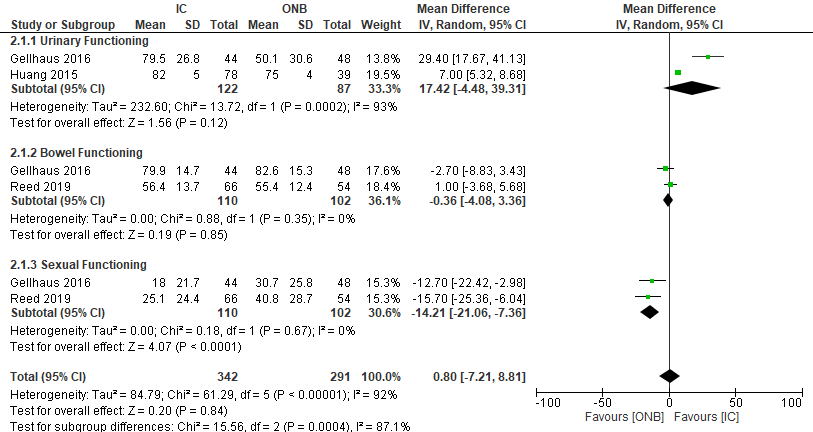


Note: The analysis compares Ileal Conduit (IC) versus Orthotopic Neobladder (ONB). Results are presented as Mean Differences (MD). Since higher scores indicate better quality of life, a negative MD value (< 0) favors the Neobladder group, while a positive MD value (> 0) favors the Ileal Conduit group. Horizontal bars denote 95% Confidence Intervals (CIs).

**6. Supplementary Figure 4:** Forest Plot for SF-36 Outcomes.


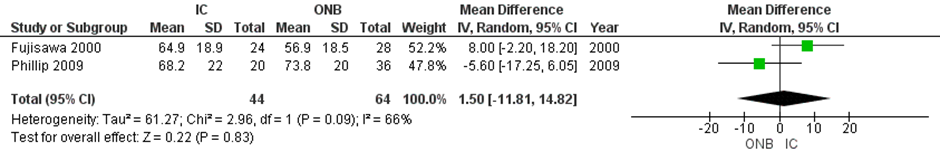


Note: The analysis compares Ileal Conduit (IC) versus Orthotopic Neobladder (ONB). Results are presented as Mean Differences (MD). Since higher scores indicate better quality of life, a negative MD value (< 0) favors the Neobladder group, while a positive MD value (> 0) favors the Ileal Conduit group. Horizontal bars denote 95% Confidence Intervals (CIs).

**7. Supplementary Figure 5a:** Forest Plot for FACT-BL Outcomes.


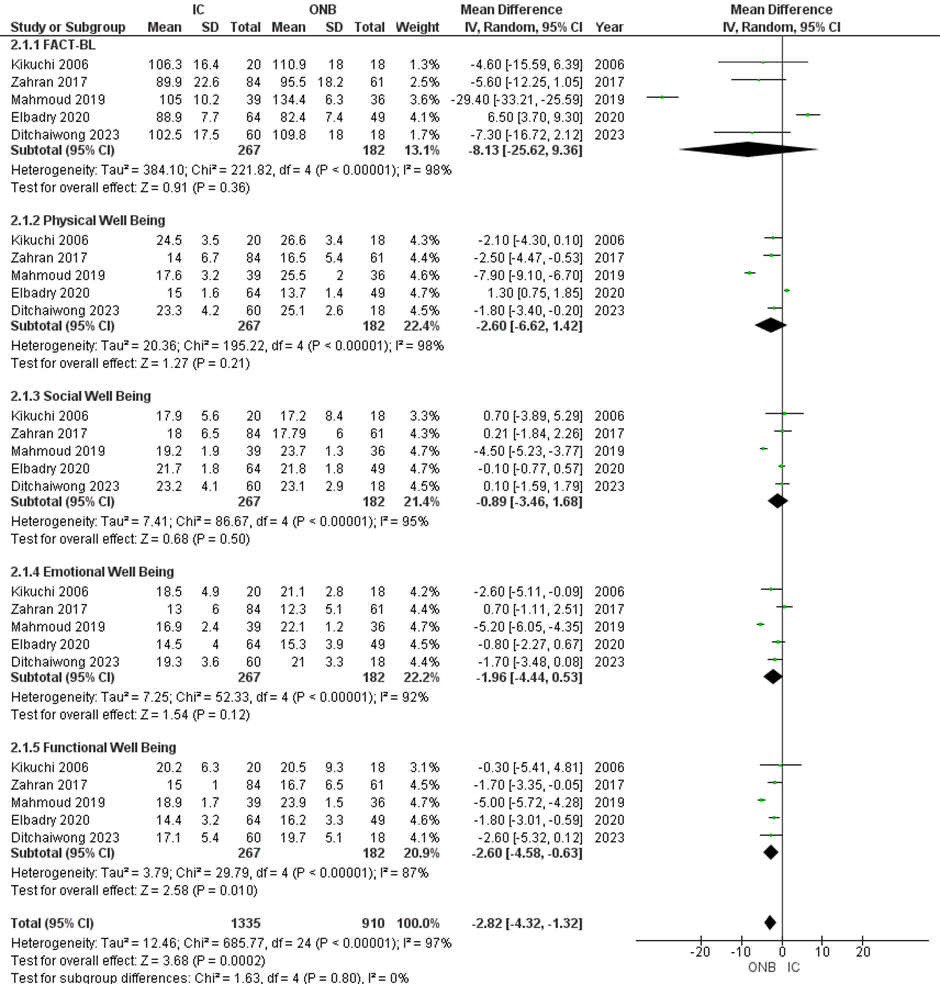


Note: The analysis compares Ileal Conduit (IC) versus Orthotopic Neobladder (ONB). Results are presented as Mean Differences (MD). Since higher scores indicate better quality of life, a negative MD value (< 0) favors the Neobladder group, while a positive MD value (> 0) favors the Ileal Conduit group. Horizontal bars denote 95% Confidence Intervals (CIs).

**8. Supplementary Figure 5b:** Forest Plot for FACT-BL After Sensitivity Analysis


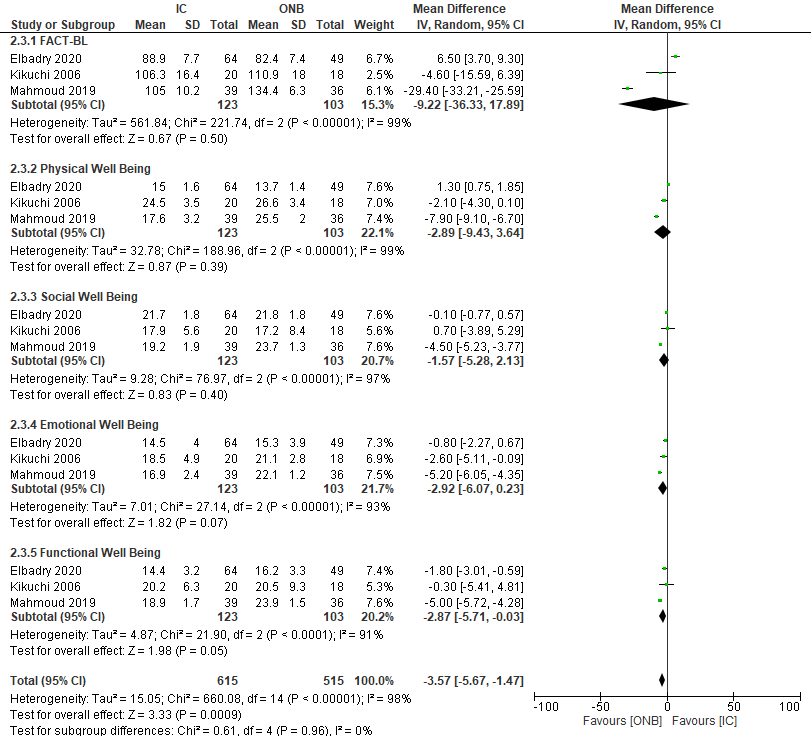


Note: The analysis compares Ileal Conduit (IC) versus Orthotopic Neobladder (ONB). Results are presented as Mean Differences (MD). Since higher scores indicate better quality of life, a negative MD value (< 0) favors the Neobladder group, while a positive MD value (> 0) favors the Ileal Conduit group. Horizontal bars denote 95% Confidence Intervals (CIs).

**9. Supplementary Table 1. Summary of Meta-Analysis**

| Subgroup | No. of studies | Sample size | | Heterogeneity | | Overall effect size | 95% CI of overall effect | P Value |
| --- | --- | --- | --- | --- | --- | --- | --- | --- |
|  |  | IC | ONB | I² (%) | P Value |  |  |  |
| EORTC-QLQ–C30 Global Health Status | | | | | | | | |
| Prospective | 2 | 127 | 120 | 28 | 0.24 | MD=-17.89 | -22.41~-13.38 | <0.00001 |
| Cross-sectional | 4 | 281 | 296 | 80 | 0.002 | MD=-4.24 | -13.33~4.85 | 0.36 |
| Retrospective | 2 | 41 | 50 | 0 | 0.85 | MD=-13.28 | -23.08~-3.48 | 0.008 |
| EORTC-QLQ–C30 Physical Functioning | | | | | | | | |
| Prospective | 2 | 120 | 127 | 92 | 0.001 | MD= -8.46 | -27.72 ~0.80 | 0.39 |
| Cross-sectional | 4 | 296 | 281 | 77 | 0.004 | MD= 2.83 | -6.52~12.19 | 0.55 |
| Retrospective | 2 | 50 | 41 | 57 | 0.13 | MD= -9.08 | -25.49~7.33 | 0.28 |
| EORTC-QLQ-C30 Emotional Functioning | | | | | | | | |
| Prospective | 2 | 120 | 127 | 0 | 0.39 | MD=-0.09 | -3.53~3.35 | 0.96 |
| Cross-sectional | 4 | 296 | 281 | 88 | 0.000 | MD=1.08 | -12.42~14.58 | 0.88 |
| Retrospective | 2 | 50 | 41 | 0 | 0.41 | MD=-4.49 | -12.46~  .3.47 | 0.27 |
| FACT-BL | | | | | | | | |
| PWB | 5 | 267 | 182 | 98 | 0.000 | MD=-2.6 | -6.62~1.42 | 0.21 |
| SWB | 5 | 267 | 182 | 95 | 0.000 | MD=-0.89 | -3.46~1.68 | 0.5 |
| EWB | 5 | 267 | 182 | 92 | 0.000 | MD=-1.96 | -4.44~0.53 | 0.12 |
| FWB | 5 | 267 | 182 | 87 | 0.000 | MD=-2.6 | -4.58~-0.63 | 0.01 |
| SF-36 | | | | | | | | |
| SF-36 | 2 | 44 | 64 | 66 | 0.09 | MD=1.5 | -11.81~14.82 | 0.83 |
| BCI | | | | | | | | |
| UF | 4 | 272 | 182 | 100 | 0.000 | MD=22.81 | 2.99~42.64 | 0.02 |
| BF | 4 | 260 | 197 | 91 | 0.000 | MD=2.86 | -2.94~8.66 | 0.33 |
| SF | 4 | 260 | 197 | 95 | 0.000 | MD=-2.23 | -15.04~10.59 | 0.73 |
