# Supplementary material for: Quality of Life After Radical Cystectomy: Meta-analysis of Neobladder and Ileal Conduit Outcomes Across Multiple Assessment Tools
Source: Eur Urol Open Sci. 2026 Apr 16;87:115–24. doi: 10.1016/j.euros.2026.03.005 (PMC13101609; doi:10.1016/j.euros.2026.03.005)
Supplement: Supplementary Data 3 [file mmc3.docx]

**Supplementry Table 2.** Search strategy used in our systematic review of quality of life after radical cystectomy and urinary diversion

| String |
| --- |
| ((((("Orthotopic neobladder") OR ("Urinary bladder substitution")) OR ("Bladder reconstruction")) OR ("Urinary reservoir")) OR ("Continent urinary diversion") OR ("Ileal neobladder") OR ("Colonic neobladder") OR ("Ileal conduit neobladder"))  AND  ((((((((("Radical cystectomy") OR ("Cystectomy"[MeSH Terms])) OR ("Total cystectomy")) OR ("Complete cystectomy")) OR ("Extirpative cystectomy")) OR ("En bloc cystectomy")) OR ("Radical bladder resection")) OR ("Total urinary bladder excision")) OR ("Radical vesical excision")))  AND  ((((((((((("Quality of life") OR ("Health-related quality of life")) OR ("Well-being")) OR ("Life satisfaction")) OR ("Functional status")) OR ("Patient-reported outcomes")) OR ("Health status")) OR ("Standard of living")) OR ("Psychosocial well-being")) OR ("General well-being")) OR ("Subjective well-being")))  AND  ((cohort) OR (retrospective)) |
| 1429 articles |
