# Supplementary material for: Quality of Life After Radical Cystectomy: Meta-analysis of Neobladder and Ileal Conduit Outcomes Across Multiple Assessment Tools
Source: Eur Urol Open Sci. 2026 Apr 16;87:115–24. doi: 10.1016/j.euros.2026.03.005 (PMC13101609; doi:10.1016/j.euros.2026.03.005)
Supplement: Supplementary Data 4 [file mmc4.docx]

Supplementary Table 3. Non-randomized studies risk of bias assessment using Newcastle-Ottawa Scale

| Study | Selection | Comparability | Outcomes |
| --- | --- | --- | --- |
| Siracusano., 2023 | *** | ** | *** |
| Siracusano., 2022 | *** | ** | ** |
| Biardeau., 2020 | ** | ** | *** |
| Kretschmer., 2019 | ** | * | ** |
| Siracusano., 2019 | ** | ** | *** |
| Cerruto., 2017 | ** | ** | *** |
| Zahran., 2017 | ** | ** | *** |
| Singh., 2013 | ** | * | *** |
| Erber., 2012 | ** | ** | *** |
| Sogni., 2008 | ** | ** | *** |
| Hobisch., 2000 | ** | * | ** |
| Gacci., 2013 | ** | * | ** |
| Elbadry., 2020 | ** | ** | *** |
| Dey., 2019 | *** | ** | *** |
| Mahmoud., 2019 | *** | * | *** |
| Kikuchi., 2006 | *** | ** | *** |
| Autorino., 2009 | ** | ** | *** |
| Phillip., 2009 | ** | * | *** |
| Dutta., 2002 | ** | ** | *** |
| Osawa., 2021 | ** | * | *** |
| Bastien., 2018 | ** | ** | *** |
| Gellhaus., 2016 | ** | * | ** |
| Goldberg., 2015 | *** | ** | ** |
| Huang., 2015 | ** | ** | ** |
