# Supplementary material for: Quality of Life After Radical Cystectomy: Meta-analysis of Neobladder and Ileal Conduit Outcomes Across Multiple Assessment Tools
Source: Eur Urol Open Sci. 2026 Apr 16;87:115–24. doi: 10.1016/j.euros.2026.03.005 (PMC13101609; doi:10.1016/j.euros.2026.03.005)
Supplement: Supplementary Data 5 [file mmc5.docx]

**Supplementary Table 4:** Details of Data Transformation.

| **Study ID** | **Outcome Domain Requiring Imputation** | **Original Reported Data Format** | **Imputation/Transformation Method** | **Reference** |
| --- | --- | --- | --- | --- |
| **Biardeau et al. (2020)** | Social Functioning (SF) | Median (Interquartile Range) | Converted to Mean (SD) | Wan et al. (2014) |
| **Zahran et al. (2017)** | EORTC QLQ-C30: Global Health Status, Physical, Role, Emotional, Cognitive, Social Functioning | Median (Range) | Converted to Mean (SD) | Wan et al. (2014) |
| **Zahran et al. (2017)** | FACT-BL: Total Score, PWB, SWB, EWB, FWB | Median (Range) | Converted to Mean (SD) | Wan et al. (2014) |
| **Goldberg et al. (2015)** | Baseline Age; BCI: Sexual Function (SF) | Median (Range) | Converted to Mean (SD) | Wan et al. (2014) |
| **Ditchaiwong et al. (2023)** | FACT-BL Domains (PWB, SWB, EWB, FWB) | Mean (SE - Standard Error) | Converted SE to SD (SD = SE x √N) | Cochrane Handbook |
| **Osawa et al. (2021)** | BCI Domains (Urinary, Bowel, Sexual) | Mean (95% CI) | Converted CI to SD | Cochrane Handbook |
