# Supplementary material for: Quality of Life After Radical Cystectomy: Meta-analysis of Neobladder and Ileal Conduit Outcomes Across Multiple Assessment Tools
Source: Eur Urol Open Sci. 2026 Apr 16;87:115–24. doi: 10.1016/j.euros.2026.03.005 (PMC13101609; doi:10.1016/j.euros.2026.03.005)
Supplement: Supplementary Data 6 [file mmc6.docx]

**Supplementary Table 5:** Scale Orientation and Interpretation.

| **Instrument** | **Full Name** | **Scale Range** | **Scale Orientation** | **Interpretation of Small Values (<)** | **Interpretation of High Values (>)** |
| --- | --- | --- | --- | --- | --- |
| **EORTC QLQ-C30** | European Organisation for Research and Treatment of Cancer QoL Questionnaire | 0 – 100 | **Higher is Better** | **Undesirable:** Indicates poor quality of life or low functional status. | **Desirable:** Indicates high quality of life and healthy functional status. |
| **BCI** | Bladder Cancer Index | 0 – 100 | **Higher is Better** | **Undesirable:** Indicates poor function (e.g., severe incontinence) or high bother (severe symptoms). | **Desirable:** Indicates excellent function (e.g., continence) or low bother (few symptoms). |
| **FACT-BL** | Functional Assessment of Cancer Therapy - Bladder Cancer | 0 – 148* | **Higher is Better** | **Undesirable:** Indicates poor health-related quality of life. | **Desirable:** Indicates excellent health-related quality of life. |
| **SF-36** | Short Form 36 Health Survey | 0 – 100 | **Higher is Better** | **Undesirable:** Indicates substantial limitation or poor health status. | **Desirable:** Indicates no limitation or favorable health status. |
